# Modeling random crawling, membrane deformation and intracellular polarity of motile amoeboid cells

**DOI:** 10.1371/journal.pone.0201977

**Published:** 2018-08-23

**Authors:** Sergio Alonso, Maike Stange, Carsten Beta

**Affiliations:** 1 Department of Physics, Universitat Politecnica de Catalunya, Barcelona, Spain; 2 Institute of Physics and Astronomy, Universität Potsdam, Potsdam, Germany; Université de Genève, SWITZERLAND

## Abstract

Amoeboid movement is one of the most widespread forms of cell motility that plays a key role in numerous biological contexts. While many aspects of this process are well investigated, the large cell-to-cell variability in the motile characteristics of an otherwise uniform population remains an open question that was largely ignored by previous models. In this article, we present a mathematical model of amoeboid motility that combines noisy bistable kinetics with a dynamic phase field for the cell shape. To capture cell-to-cell variability, we introduce a single parameter for tuning the balance between polarity formation and intracellular noise. We compare numerical simulations of our model to experiments with the social amoeba *Dictyostelium discoideum*. Despite the simple structure of our model, we found close agreement with the experimental results for the center-of-mass motion as well as for the evolution of the cell shape and the overall intracellular patterns. We thus conjecture that the building blocks of our model capture essential features of amoeboid motility and may serve as a starting point for more detailed descriptions of cell motion in chemical gradients and confined environments.

## Introduction

The ability of cells to move is an essential prerequisite for a wide range of biological processes including wound healing, morphogenesis, and cancer progression [1]. Frequently, motile cells define a *head* and a *tail*, to decide in which direction to move in a persistent fashion. Such processes of intracellular symmetry breaking are known as cell polarization [2]. Cell polarity emerges not only in migrating cells but is observed in many different contexts such as asymmetric cell division and directed tissue growth [3]. It has been computationally modeled at different spatial scales and under different levels of approximation for a wide range of cell types, see for example [4–6] and references therein.

A particularly well-suited system to study the role of polarity during cell locomotion is the social amoeba *Dictyostelium discoideum*, which has become a well-established model organism for the study of chemotaxis [7]. In the absence of chemoattractant gradients, *Dictyostelium* cells migrate spontaneously based on correlated deformations of their shape [8]. When exposed to a nonuniform chemoattractant profile, they bias their motion towards increasing chemoattractant concentrations. In this case, the variety of amoeboid cell shapes has also been attributed to strategies of accurate gradient sensing [9]. Prominent features of the cell shape dynamics are localized protrusions that are called pseudopods and can be considered the basic stepping units of amoeboid motion [10]. The ordered appearance of pseudopods and their biased formation in the presence of a chemoattractant gradient form the basis of persistent amoeboid motion [11, 12] and have inspired the use of random stepping models for mathematical descriptions of cell trajectories [13]. The resulting center-of-mass motion can be also described in terms of stochastic differential equations derived directly from the experimentally recorded trajectories [14–17]. These approaches were extended to biased random movement in a chemoattractant gradient [18] and highlight non-Brownian features of *Dictyostelium* locomotion [19].

Depending on the nutrient conditions, *Dictyostelium* may enter a developmental cycle that stronlgy affects cell speed and polarity. If food is abundant, cells remain in the vegetative state that is characterized by slow apolar motion, where pseudopods are formed in random directions. If food becomes sparse, a developmental cycle is initiated that ultimately leads to the formation of a multicellular fruiting structure. In the beginning, over the first hours of starvation-induced development, cells become chemotactic to cAMP, the speed increases, and cell movement becomes increasingly polar with pseudopods preferentially forming at a well-defined leading edge [20].

From experiments with fluorescently labeled *Dictyostelium* constructs it is well known that under the influence of a chemoattractant gradient, a polar rearrangement of various intracellular signaling molecules and cytoskeletal components can be observed [21]. For example, the phospholipid PIP_3_ accumulates at the membrane in the front part of the cell, while at the sides and in the back predominantly PIP_2_ is found [22]. Consequently, also the PI3-kinase that phosphorylates PIP_2_ to PIP_3_ and the phosphatase PTEN that dephosphorylates PIP_3_ are polarly distributed along the cell membrane. Similarly, also the downstream cytoskeletal network exhibits a polar arrangement with freshly polymerized actin and the Arp2/3 complex at the leading edge, while the sides and back are enriched in myosin II. Also more complex patterns are observed, such as waves and oscillatory structures that emerge at different levels of the signaling system and the actin cytoskeleton [23–26]. Note that similar processes are also responsible for cell polarization and locomotion of neutrophils, which are highly motile white blood cells [27].

A variety of mathematical models have been proposed to rationalize the mechanisms of gradient sending, polarization, and locomotion of *Dictyostelium*. In general, these models consider the dynamics of the concentrations of some of the above mentioned biochemical species at different levels of detail [28, 29]. They are typically of reaction-diffusion type and give rise to instabilities that result in the formation of complex intracellular patterns, see for example [30–32]. In combination with the local excitation global inhibition mechanisms of directional sensing [33, 34], several modeling variants have been proposed that describe the rich dynamics of intracellular patches, oscillations, and waves in motile cells [35]. In order to account for cell shape dynamics and motility, the intracellular processes are coupled to deformations of a bounded domain. Here, a particularly popular approach is the use of an auxiliary phase field to mimic the presence of a cell membrane. The phase field method has been recently used to model amoeboid motion [32, 36, 37], following previous successful applications to other cell types [38–41]. However, besides the use of a phase field, also other approaches have been used, such as level set methods, see for example [42]. In these models, pseudopods are typically initiated by a noisy excitable system, where the excitation threshold may depend on external gradient stimuli and can be modulated by an additional polarity module [42–44]. Overall, these approaches are driven by the motivation to include as many of the known biochemical details as possible into the design of the model. However, they largely neglect the inherent cell-to-cell variability that is typically encountered when analyzing the motile properties of an otherwise uniform cell population.

In the present work, we take a different perspective and ask for the minimal number of essential ingredients needed in order to capture the phenomenology of amoeboid cell trajectories and shape dynamics. We propose a reduced model that is based on the interplay of a highly stochastic, protrusive membrane activity and a slowly varying deterministic polarity mechanism coupled to a phase field in order to describe the shape and the deformations of the membrane. In particular, our model is designed to account for variability in the motile properties of amoeboid cells by including a parameter that tunes the balance between the mechanism of polarity formation and intracellular noise.

## Materials and methods

### Experimental setup

#### Cell culture

*Dictyostelium discoideum* Ax-2 cells expressing a fluorescently labeled version of the Lifeact protein (C-terminally tagged with mRFP) as a marker for filamentous actin were grown in liquid culture flasks (HL5 media including glucose, Formedium, Hunstanton, UK) containing the required selection marker (G-418 sulfate: Cayman Chemical Company, USA, 10 *μ*g/ml) at 20°C. The Lifeact encoding plasmid was kindly provided by the lab of Igor Weber, Zagreb. Prior to experiments, cells were harvested and cultivated over night in a 25 ml shaking culture at 180 rpm under the same conditions. The cell suspension was centrifuged and the cell pellet washed with Sørensen phosphate buffer at pH6 (KH_2_PO_4_, 14.7 mM, Merck, Darmstadt, GER; Na_2_HPO_4_*2H_2_O, 2 mM, Merck, Darmstadt, GER) to remove all nutrients. Cells were resuspended in fresh buffer and droplets were formed in a Petri dish to initiate the streaming processes.

#### Image acquisition

After 5 hours, cells were transferred to a glass bottom dish (Fluorodish, ibidi GmbH, Martinsried, GER) and constantly kept in Sørensen phosphate buffer at 20°C during imaging. Laser scanning microscopy was performed with a confocal Zeiss LSM780 microscopy system (Carl Zeiss AG, Oberkochen, GER) with a 63x or 40x magnification oil-immersion objective. We record the images with a frequency of one frame per second. Fluorescence was excited at 651 nm and emission detected between 562 nm and 704 nm. ZEN-software recommended by Zeiss was used for microscope settings and recording.

#### Image processing

Image sequences (8-bit gray scale) were analyzed using a modified version of the active contour algorithm described in [45, 46]. For each frame the cellular boundary was parameterized by a string of 400 nodes, which were tracked over time by using a least square mapping. In this way, the change in cell shape, the local motion of each node, as well as the intensity of the actin marker at the boundary were determined and displayed in kymographs.

### Mathematical model

We model the processes in the interior of the cell and the deformation of the cell membrane. The membrane defines the domain of integration of the internal processes. Thus, a crawling cell is a moving boundary problem, which is mathematically complicated to solve. A typical approach for such moving boundary problems is the use of an auxiliary field which keeps the non-flux boundary conditions at the border and evolves with the shape of the domain. This approach has been previously employed also in other fields to describe, for example, solidification [47], fracture of solids [48], or fluid-fluid interfaces [49]. Here we employ a finite differences method with Δx = 0.15 *μ*m and Δt = 0.002 s. Specific values of the parameters are shown in Table 1.

**Table 1:** Parameter values and meaning.

Parameter	Value	Meaning
*τ*	2.0 pN s *μ*m^−2^	time scale of membrane dynamics
*γ*	2.0 pN	surface tension
*ϵ*	0.75 *μm*	interface width
*β*	22.2 pN *μ*m^−3^	total area constraint parameter
*A*_*o*_	113 *μ*m^2^	area of the cell
*α*	3 pN *μ*m^−1^	active tension
*k*_*a*_	2-5 *s*^−1^	reaction rate
*δ*_*o*_	0.5	bistability parameter
*C*_*o*_	28 *μ*m^2^	Maximum area coverage by *c*
*M*	0.045 *μ*m^−2^	constraint parameter
*ρ*	0.1 *s*^−1^	degradation time
*D*	0.5 *μm*^2^/*s*	diffusion coefficient
*σ*	0.15 *s*^−2^	white noise intensity
*k*_*η*_	0.1 *s*^−1^	Orsntein-Uhlembeck rate

List of the parameters employed in the modeling and the corresponding values used in the numerical simulations.

#### Phase field

The phase field $\phi (\vec{x})$ defines the area of the cell and it is *ϕ* = 1 inside and *ϕ* = 0 outside the cell. The transition between these two values is smooth at the membrane which is defined by the value *ϕ* = 0.5. The evolution of the phase field follows the integro-differential equation

$$\begin{array}{ccc} \tau \frac{\partial \phi }{\partial t} & = & \gamma (\nabla^{2}\phi -\frac{G^{\prime }\left(\phi \right)}{\epsilon^{2}})-\beta (\int \phi dx-A_{o})\left|\nabla \phi \right|+\alpha c|\nabla \phi |. \end{array}$$ (1)

Its evolution is controlled by three different contributions represented by the three terms on the right hand side of Eq (1). The first one affects the whole volume while the others only affect the border of the cell, determined by the gradient |∇*ϕ*|. The specific meaning of each term is:

1. **Surface tension**: The first term can be derived from free energy arguments, where the parameter *γ* corresponds to the surface tension. The surface energy is proportional to the perimeter of the cell *L* and can be implemented in the phase-field formulations [38]:
  $$\begin{array}{c} H_{ten}=\gamma L=\int (\frac{1}{2}{\left|\nabla \phi \right|}^{2}+\frac{G(\phi )}{\epsilon^{2}}) \end{array}$$ (2)
  where *ϵ* is the parameter controlling the width of the cell boundary and *G*(*ϕ*) is a double well potential: *G*(*ϕ*) = 18*ϕ*^2^(1 − *ϕ*)^2^. It corresponds to the force, see [50]:
  $$\begin{array}{c} F_{ten}=-\gamma (\nabla^{2}\phi -\frac{G^{\prime }\left(\phi \right)}{\epsilon^{2}})\frac{\nabla \phi }{{\left|\nabla \phi \right|}^{2}}. \end{array}$$ (3)
  If we consider only this term in 1D systems, the evolution of Eq (1) produces stationary profiles. However, in 2D the total volume of the cell decreases with time due to the surface tension.
2. **Volume conservation**: The term proportional to *β* accounts for the cell volume conservation. It decreases or increases the size of the cell if the volume is, respectively, larger or smaller than the prescribed volume *A*_*o*_. This integral, together with the previous term, ensures the existence of circular domains in 2D systems with a volume close to *A*_*o*_ and is equivalent to the force:
  $$\begin{array}{c} F_{area}=\beta (\int \phi dx-A_{o})\frac{\nabla \phi }{\left|\nabla \phi \right|}. \end{array}$$ (4)
3. **Active tension**: The term proportional to *α* accounts for the force generated by the cell to produce the directed motion following the pattern of the biochemical component *c* formed in the interior of the cell. The force of the cell is proportional to the parameter *α*:
  $$\begin{array}{c} F_{act}=-\alpha c\frac{\nabla \phi }{\left|\nabla \phi \right|}. \end{array}$$ (5)

Together with the friction of the cell with the substrate (F_*fric*_ = −*τv*), the forces satisfy the condition F_*tot*_ = 0 at the quasi-steady state, which results in ∂_*t*_*ϕ* = −*v* ⋅ ∇*ϕ* giving rise to Eq (1), see [50] for a complete derivation.

#### Biochemical reaction-diffusion process

Inside the cell there is a reaction-diffusion process governed by a biochemical component *c*. This component reacts and diffuses inside the cell. The concentration of *c* evolves according to the following reaction-diffusion equation,

$$\begin{array}{ccc} \frac{\partial c}{\partial t} & = & k_{a}c\left(1-c\right)\left(c-\delta \right)-\rho c+\frac{1}{\phi }\vec{\nabla }\cdot \left(\phi D\vec{\nabla }c\right)+\xi \left(\vec{x},t\right)\phi \left(1-\phi \right), \end{array}$$ (6)

The concentration c accounts for different subcellular factors, which promote the growth of filamentous actin and thus the formation of pseudopods. It corresponds to the combined effective action of signaling components such as activated Ras, PI3K, or PIP_3_, which are known to enhance the formation of F-actin [21]. For this reason a fluorescent F-actin marker is the appropriate experimental readout to compare with the action of the component c.

1. **Cell polarity**: Cell polarity has been modeled in a variety of different ways [2]. In the case of *Dictyostelium*, noisy excitable systems with a slow diffusing polarity component have been favored recently and were compared with experimental data in detail [42–44]. In these models, larger values of the polarity marker lower the excitation threshold, so that the signaling system is more likely to switch to the excited state. Here, we reduce this description to a single species c that can switch between a passive (low concentration of c) and an active state (high concentration of c) in a bistable fashion. We therefore chose a generic bistable kinetics for the component c, reflected in the first term on the right hand side of Eq (6). Thus, the form of this term does not correspond to a specific biochemical reaction in the cell but should be rather seen as an effective model of the overall kinetics of the intracellular polarity markers. In the case of *Dictyostelium*, it is well known that the signaling pathway involving Ras, PI3K and F-actin involves positive feedback loops that can lead to nonlinear behavior [51]. Note also that in other cell types, such as mesenchymal breast cancer cells, bistable kinetics has been observed in actin-related signaling networks [52].
  $$\begin{array}{c} \delta =\delta_{o}+M(\int c\phi dx-C_{o}). \end{array}$$ (7)
  This integral term produces a constant global concentration of *c* in the whole cell *C*_*o*_, occupying a quarter of the cell size. The concentration of *c* tends to accumulate and to produce domains due to the nonlinear reaction in Eq (6).
2. **Linear degradation**: The biochemical processes eventually degrade the species *c* and if there is no reaction the concentration of *c* decreases to zero.
3. **Diffusion**: The biochemical species *c* diffuses freely inside the cell, however, the presence of the phase field produces a no-flux boundary condition at the membrane of the cell.
4. **Spatio-temporal noise**: Inside the cell, stochastic fluctuations are relevant and may determine the final behavior of the cells. Here, we model this stochastic contribution by a random component in the dynamics of the biochemical component c that we confine to the cortical region of the cell, where most of the relevant signaling events take place. The stochastic term follows an Ornstein-Uhlembeck dynamics:
  $$\begin{array}{c} \frac{\partial \xi }{\partial t}=-k_{\eta }\xi +\eta , \end{array}$$ (8)
  where *η* is a Gaussian white noise with zero mean average 〈*η*〉 = 0 and variance 〈*η*(*x*, *t*)*η*(*x*′, *t*′)〉 = 2*σ*^2^*δ*(*x* − *x*′)*δ*(*t* − *t*′).

## Results

### A model that encodes variability in the motion of *Dictyostelium discoideum*

**Fig 1:**
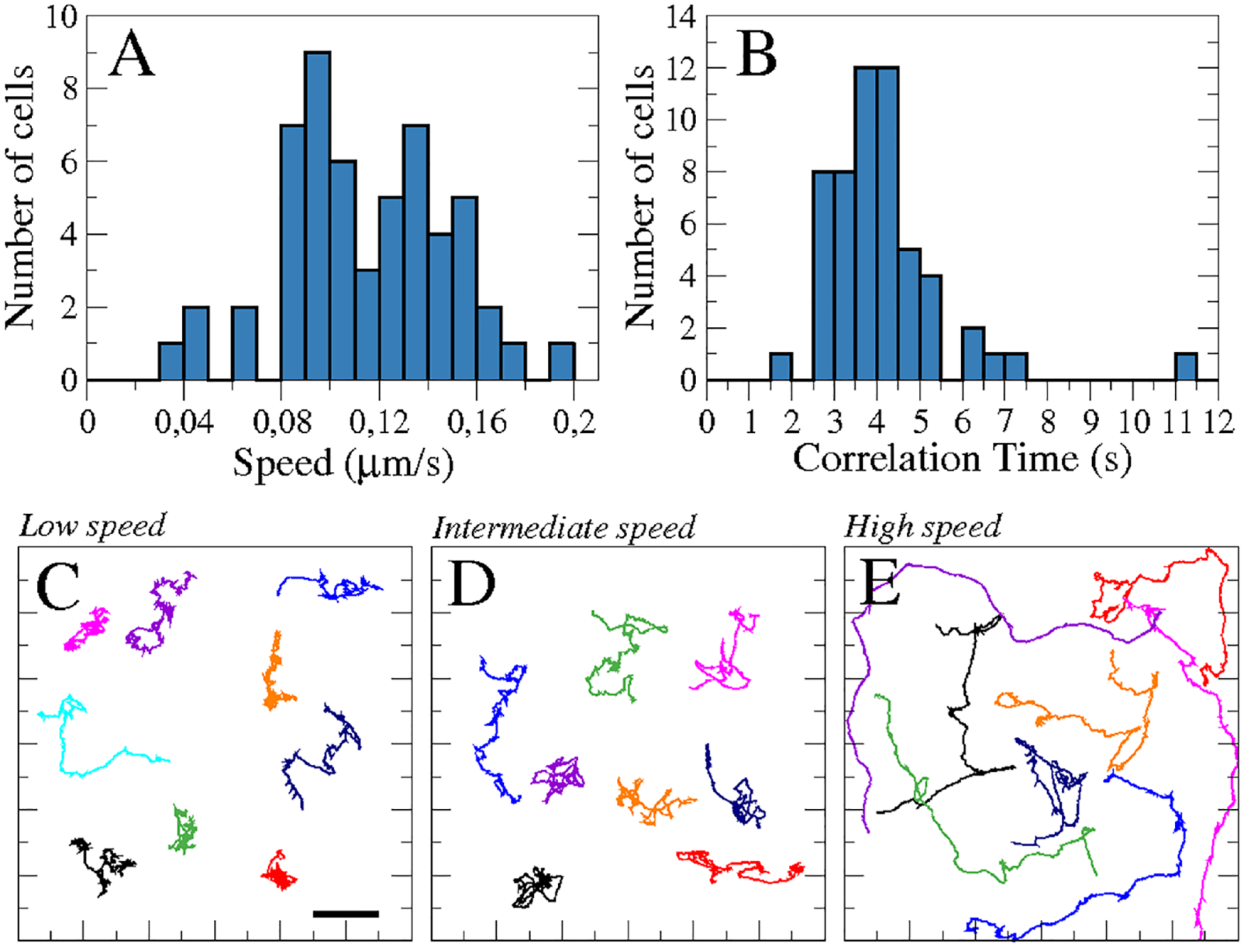
Dynamics of a population of 55 cells. Histogram of the averaged speed of the cells (A), and the characteristic correlation time (B). Individual trajectories of cells classified by their averaged speed: Low, intermediate, and high speed corresponding, respectively, to 0.09-010 *μ*m/s (N = 9 cells) (C), 0.12-013 *μ*m/s (N = 8 cells) (D), and 0.15-019 *μ*m/s (N = 8 cells) (E). Panels (C), (D), and (E) have the same spatial scales. Scale bar in panel (C) corresponds to 10 *μ*m.

In the absence of sources of chemoattractant, *Dictyostelium discoideum* cells randomly inspect the surrounding space in search of food. Upon starvation, they enter a developmental cycle which will, over the first hours of development, alter their motility from slow random movements to faster and increasingly persistent locomotion. However, despite their uniform origin, development-induced changes in the motile properties do not emerge uniformly across a population of cells. This may be caused by variations in the cell micro-environment during development, such as fluctuations in the local cell density or in cell-cell signaling and contacts [53]. Cells can show diverse averaged speeds, see Fig 1(A) for an example, where a histogram of the average speeds of 55 different cells with a mean speed of the population of 〈*v*〉 = 0.11 ± 0.03 *μ*m/s is shown. Also other movement properties show pronounced variations across the cell population. For example the characteristic autocorrelation time *τ*_*a*_, which is the time over which speed correlations have decayed by 50%, spread over a wide range with an average value of 〈*τ*_*a*_〉 = 4.0 ± 1.0 s, see Fig 1(B). Examples of the diversity of cell trajectories are shown in Fig 1(C)–1(E).

**Fig 2:**
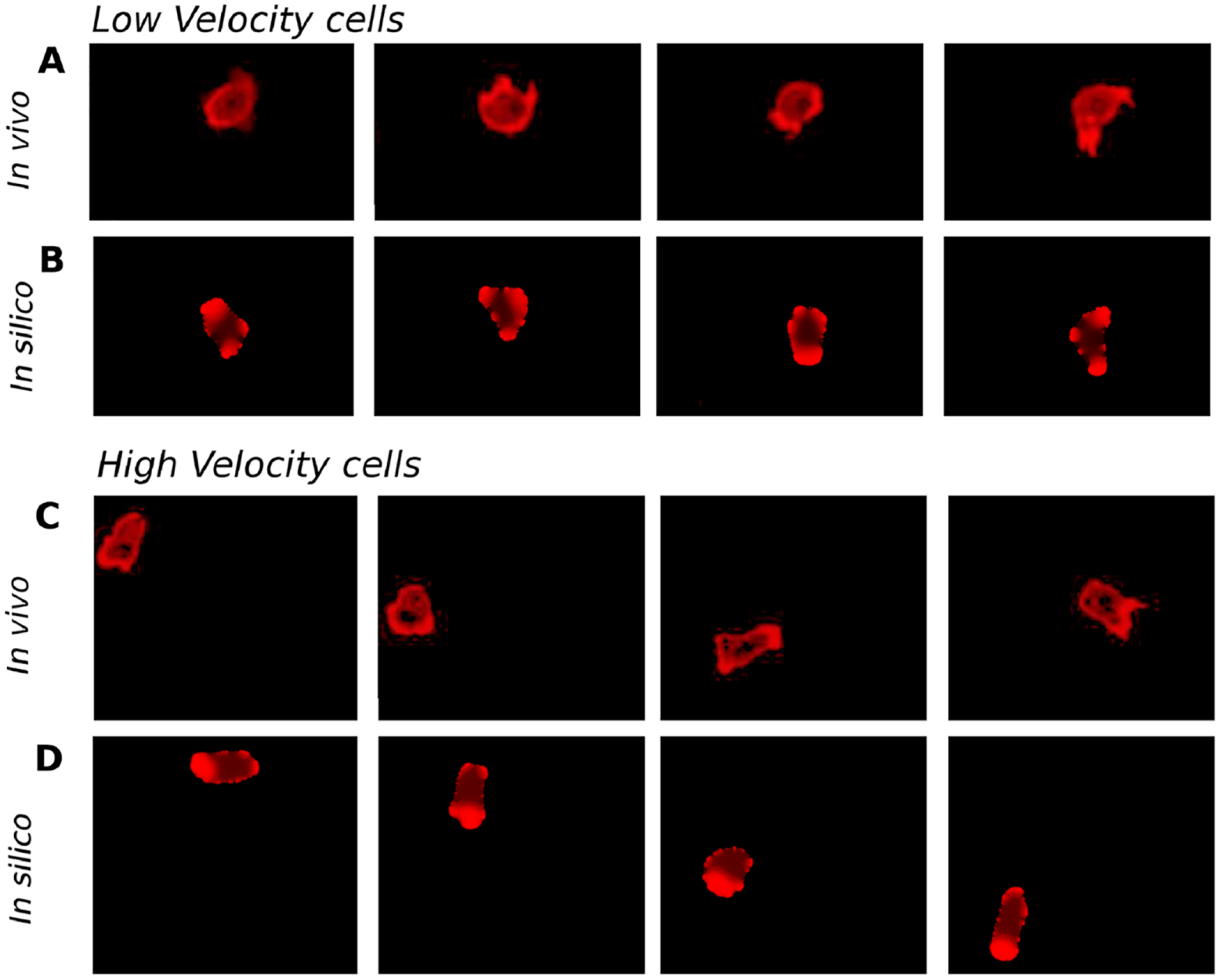
Cell dynamics in experiments and in the numerical simulations. Sequence of four snapshots of cell motion, taken every 100 seconds, for low speed in experiments (A), for low speed in the computer simulations with parameter *k*_*a*_ = 2 (B), for high speed in experiments (C), and for high speed in the computer simulations with parameter *k*_*a*_ = 5 (D). Red color is proportional to the concentration of actin in experiments (A,C) and to the concentration *c* resulting from Eq (6) in computer simulations (B,D).

In Fig 2, snapshots of two representative examples from the slow and the fast moving subpopulations are shown, see also S1–S4 Videos. While the first cell performs short random movements around its initial location, see Fig 2(A), the second cell, even though it has starved for the same time, moves much faster and explores space by more persistent motion, see Fig 2(C). This type of locomotion is associated with a persistent accumulation of freshly polymerized actin at the membrane of the leading edge.

In our numerical model that was introduced in the previous section, the transition from slow erratic motility to fast and persistent motion is encoded in the parameter *k*_*a*_ of Eq (6). This parameter controls the impact of the bistable pattern formation mechanism compared to the random displacements produced by the spatio-temporal noise $\xi (\vec{x},t)$. As the noise intensity is kept constant for all the numerical simulations, a change in *k*_*a*_ determines to what extent the first term in Eq (6), which is responsible for polarization of the cell, dominates the motile behavior given the constant noise level. By tuning *k*_*a*_, our model permits a well-controlled and continuous transition from an apolar state, characteristic for vegetative cells, to a highly polarized condition, typically encountered for starvation-developed cells. We hypothesize that cells may have different rates at which *k*_*a*_ grows with starvation time. Also, *k*_*a*_ may depend on other factors, such as cell density, cell-cell signaling, and other influences from the cell micro-environment. Overall, we employ different values of the parameter *k*_*a*_ to model heterogeneity in the motile properties of starvation-developed *Dictyostelium* cells.

In Fig 2(B) and 2(D), we show the results of numerical integration of Eqs (1), (6) and (8) for two different values of *k*_*a*_, see additional examples in S5–S8 Videos. While for *k*_*a*_ = 2 the motion is restricted to a small region of space and is characterized by a continuous generation and disappearance of protrusions in random directions, see Fig 2(B), for *k*_*a*_ = 5 the simulated cell explores larger regions of space and a persistent accumulation of c is located in the direction of motion, see Fig 2(D). Note that although we compare the results on the dynamics of the field c in our numerical simulations with the dynamics of actin in the living cells, the component c does not directly correspond to actin. It rather represents the combined action of upstream signaling components that were observed to promote actin polarization in *Dictyostelium discoideum*, so that the F-actin concentration is the best-suited experimental observable to compare with the action of c. We will now characterize these different motile states by analyzing the center of mass motion and the dynamics of the cell shape in more detail.

### Variability in the center-of-mass motion

#### Cell trajectories

**Fig 3:**
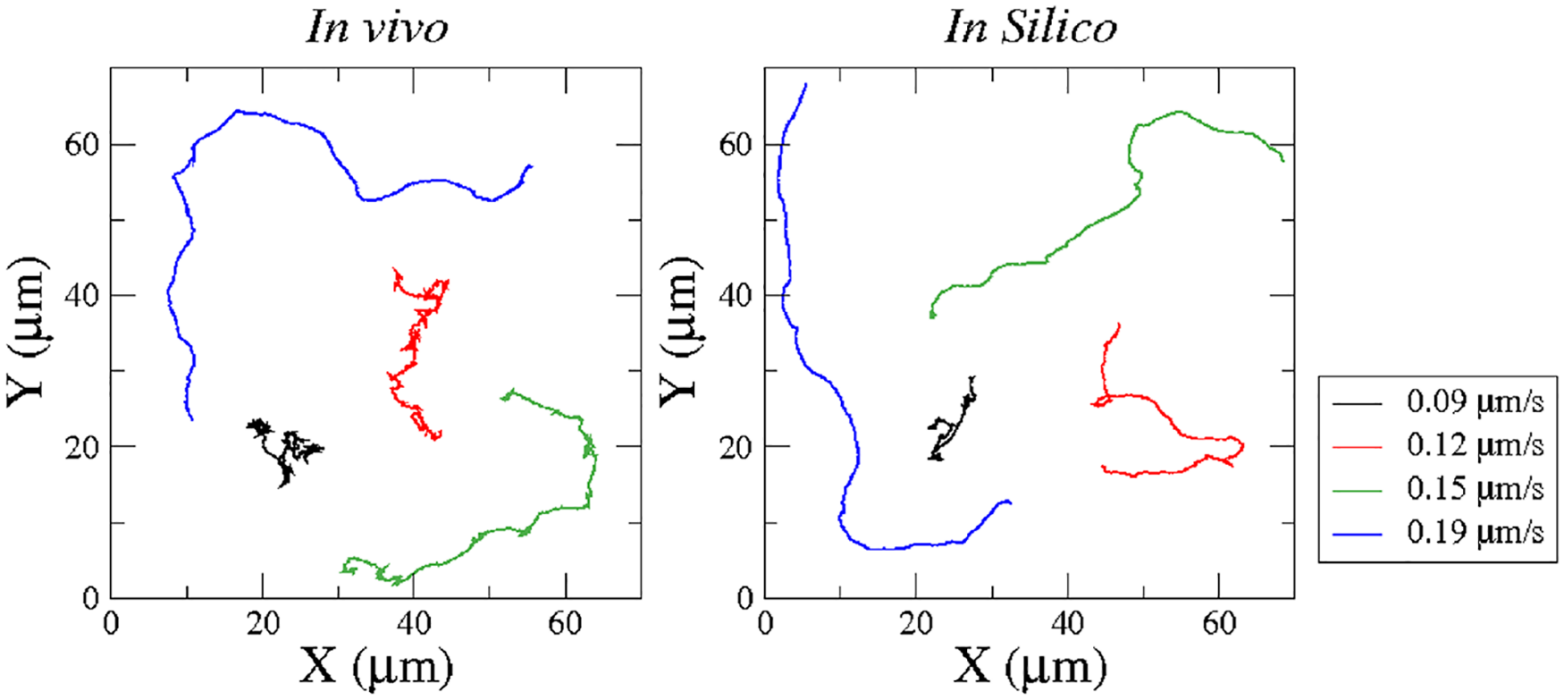
Trajectories of the center of mass of four cells with different average speeds. Four different cells from the experiment are tracked and the resulting center of mass motion is plotted over 500 *s*. Four different numerical simulations of cell motion are performed and the center of mass motion is calculated and plotted over 500 *s*. The four numerical simulations are done with the parameter values *k*_*a*_ = 2, *k*_*a*_ = 3, *k*_*a*_ = 4, and *k*_*a*_ = 5 corresponding to the average speeds used in the figure legend.

The trajectory of a cell’s center and its instantaneous velocity can be determined by finding the center of mass of a cell in each experimental image, see for example Fig 2(A) and 2(C). As indicated in the previous section, the shapes of the trajectories may vary strongly from one cell to another. Several characteristic examples are shown in Fig 3(A). Slow moving cells produce a fluctuating pattern, where the direction of motion changes frequently, while faster cells may propagate in a more persistent fashion. Note that in general we have to distinguish cell velocities calculated on short and long time scales. While the short timescale velocity (“instantaneous velocity”) is dominated by noise and irregular fluctuations of the cell shape, studies of persistent cell motion typically employ averaged velocities, where such fluctuations are filtered by calculating the velocity on larger time scales, see for example [17].

The same cell tracking algorithms that were applied to process the experimental data can be also used for the analysis of the numerical simulations, see Fig 3(B) and 3(D) for examples of the numerical results. While an erratic, fluctuating motion is typical for small values of the model parameter *k*_*a*_, large persistent displacements are observed in numerical simulations with larger values of *k*_*a*_, see Fig 3(B) for examples of computer generated trajectories with the respective parameter values given in the caption. We find good qualitative agreement of the experimental and numerical trajectories in the entire range of velocities. For large cell speeds of *v* = 0.15 *μm*/*s* and *v* = 0.19 *μm*/*s*, the agreement is remarkable, while in the cases of lower speed, the center of mass moves more erratically in the experimental cases.

#### Diffusion of cells

**Fig 4:**
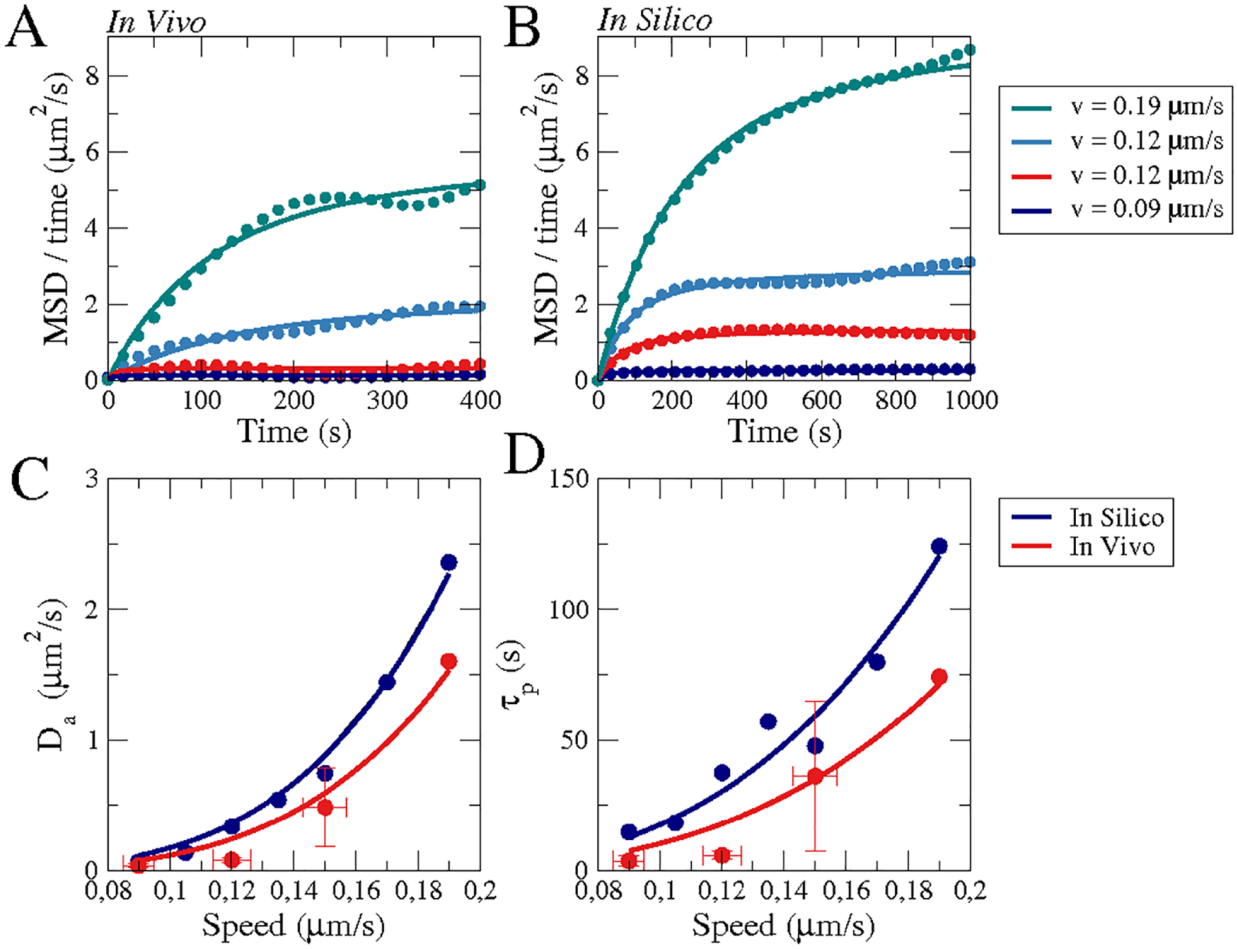
Averaged cell motion in experiments and numerical simulations. The mean square displacement divided by the time during the whole motion of four cells (A) with different averaged velocities, see legend, and for four numerical simulations (B) for four different values of *k*_*a*_ = 2 − 5 corresponding to the velocities in the legend. Solid lines (A,B) correspond to fits of Fürth’s formula for the MSD. We extract the diffusion coefficients of cells (C) and the persistent time (D) from the fitting of Fürth’s formula to the motion of 23 cells (grouped in four sets depending on the average velocity: *v* = 0.09 (N = 8 cells), *v* = 0.12 (N = 8 cells), *v* = 0.15 (N = 7 cells), *v* = 0.19 (N = 1 cells)) and compared with the fitting to 7 large computer simulations (10000 *s*) for the corresponding velocities. Fits in (C) and (D) are to guide the eye.

The present data was recorded to analyze the dynamics of the cell shape and not with the intention of producing long-time statistics for the analysis of cell trajectories, which has already been done earlier by others, see e.g. [54–56]. For this reason, our frame rate is much higher and the total time of recording much shorter than in these earlier references. Nevertheless, to perform a rough comparison, we calculated the mean square displacement (MSD = < *r*(*t*)*r*(*t* + *T*) >) of the cells to estimate their diffusive behavior. In Fig 4(a), MSD/T is shown as a function of time for four different cells. We compare these results with Fürth’s formula for the diffusion of a persistent random walker $<r\left(t\right)r\left(t+T\right)>=4D_{a}\left(T-\tau_{P}\left(1-e^{-T/\tau_{P}}\right)\right)$, where *τ*_*P*_ is the persistence time, as has been previously done for *Dictyostelium* discoideum [54, 56]. Equivalently, we also plotted and analyzed the trajectores produced by the numerical simulations, see Fig 4(b). The fit of Fürth’s formula permits to extract values for the diffusion coefficient of the cells (*D*_*a*_), see Fig 4(c), and the persistent time, see Fig 4(d), for experimental and numerical results as a function of the averaged velocities. The values of the averaged diffusion coefficient (< *D*_*a*_ > = 0.15 *μm*^2^/*s*) and the persistence time (< *τ*_*p*_ > = 26.5 *s*) are of the same order as reported earlier, albeit moderately smaller, see discussion section for more details. Note also that the persistence time is not identical to the characteristic speed correlation time described below, which is much shorter due to the strong speed fluctuations caused by the high frame rate of our recordings.

#### Cell speed and fluctuations

**Fig 5:**
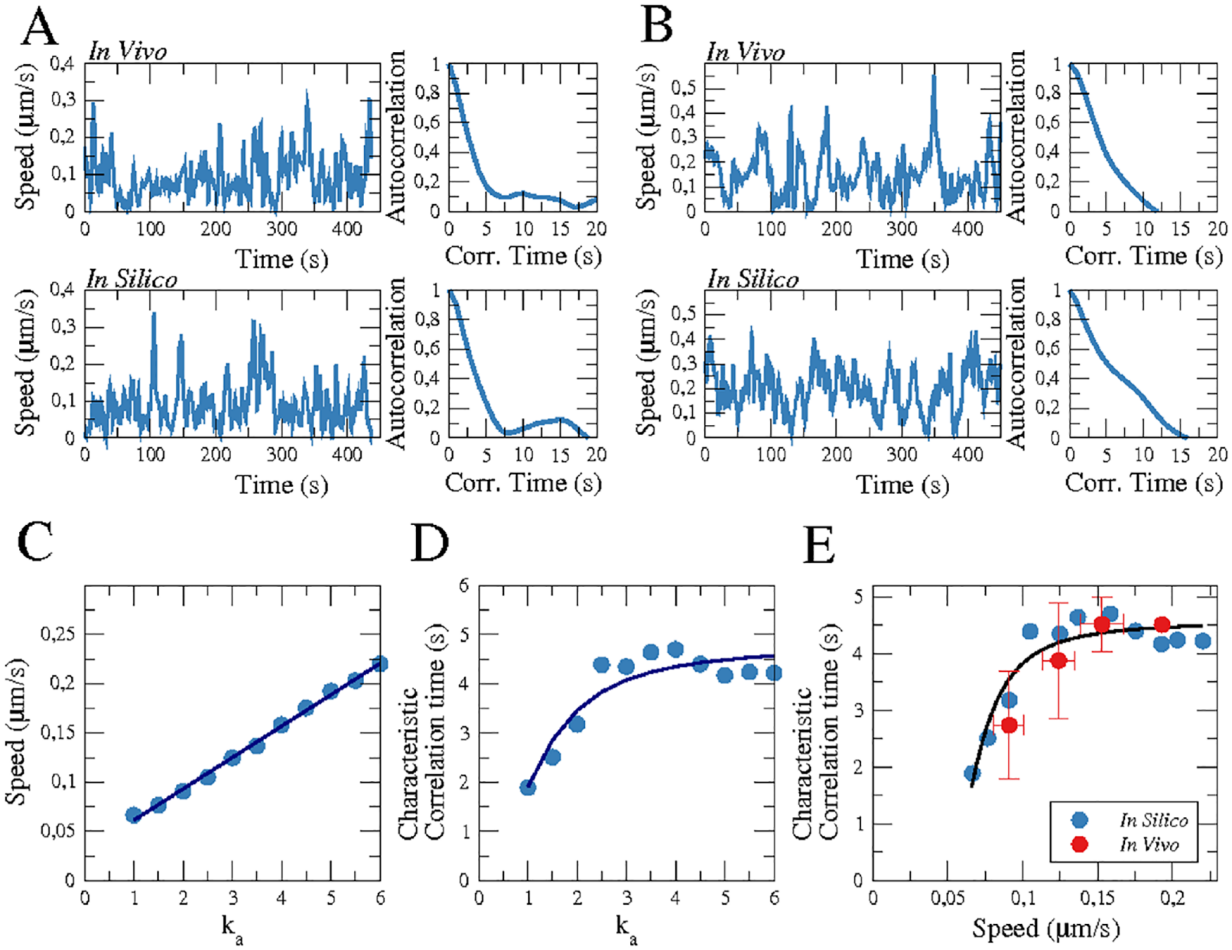
Speed and temporal correlation. Comparison of *in vivo* and *in silico* instantaneous speeds of the center of mass of the cell and the corresponding temporal autocorrelation function for cells with an average speed of 0.09 *μm*/*s* (*k*_*a*_ = 2) (A) and 0.15 *μm*/*s* (*k*_*a*_ = 5) (B). Dependence of the average speed (C) and of the characteristic correlation time (D) on the parameter *k*_*a*_, where points correspond to the resulting values and lines to fits to guide the eye. (E) Relation between cell speed and characteristic correlation time for experiments (red) and simulations (blue).

The speed of the center of mass of the cells is undergoing rapid changes over time. In Fig 5(A) and 5(B), the temporal evolution of the spontaneous speed is compared for two representative examples from the experiments and the numerical simulations. We show first the speed of a slow moving non-polar cell in Fig 5(A), which corresponds to the motion displayed Fig 2(A), and second, the speed of a fast moving polarized cell in Fig 5(B), corresponding to the more ballistic motion shown Fig 2(C). We calculate the average speeds from the temporal evolution of the speed for both living cells and for the numerical simulations. For our model we find that the speed depends linearly on the parameter *k*_*a*_, see the linear fit in Fig 5(C). For choices of *k*_*a*_ that reproduce the experimentally observed averaged cell speeds, the numerical time traces yield good agreement with the experimental results see Fig 5(A) and 5(B).

#### Autocorrelation function of cell speed

Apart from the average speed, we also calculated the autocorrelation functions of the temporal evolution of the cell speeds plotted in Fig 5(A) and 5(B). The resulting autocorrelation functions are plotted on the right hand side of the respective speed time traces. Starting from a value of 1 for a correlation time of zero seconds, the correlation function gradually decreases with increasing time shift because of the stochasticity of the speed trace. Eventually, coherence is lost and the autocorrelation function fluctuates around zero. To quantify the decrease in temporal correlation, we define the characteristic autocorrelation time (*τ*_*a*_) as the time at which the correlation function decays to 0.5. In Fig 5(D), we display the characteristic autocorrelation time as a function of the model parameter *k*_*a*_ determined from our numerical simulations. Although the average speed continuously grows with *k*_*a*_, see Fig 5(C), the value of *τ*_*a*_ saturates for large values of *k*_*a*_.

For the experimental data, there is no parameter directly accessible that corresponds to the model parameter *k*_*a*_. However, the linear relationship between the average cell speed and the model parameter *k*_*a*_ suggests that we can uniquely assign a characteristic correlation time *τ*_*a*_ to each speed value. For the model, we thus obtain a saturation curve similar to the one displayed in Fig 5(D) when plotting the characteristic correlation time as a function of the average cell speed, see the blue data points in Fig 5(E). This relation can now be compared to experimental data, as both the characteristic correlation time and the average cell speed are readily accessible from our imaging data. We determined the mean characteristic correlation time for several cells with similar average speeds and compare the resulting data points (red symbols) to the relation between *τ*_*a*_ and cell speed from our model, see Fig 5(E). We obtain a qualitative agreement between both cases. Note that no averaging was performed for the cell velocity, so that the rapid decay in the autocorrelation function at short times mostly reflects the decaying correlations in the fluctuating short timescale velocity. This is not related to the characteristic directional persistence time of cell motion that typically results in a slow decrease of the velocity autocorrelations at longer time scales, see for example [17].

#### Non-Gaussian velocity distributions

**Fig 6:**
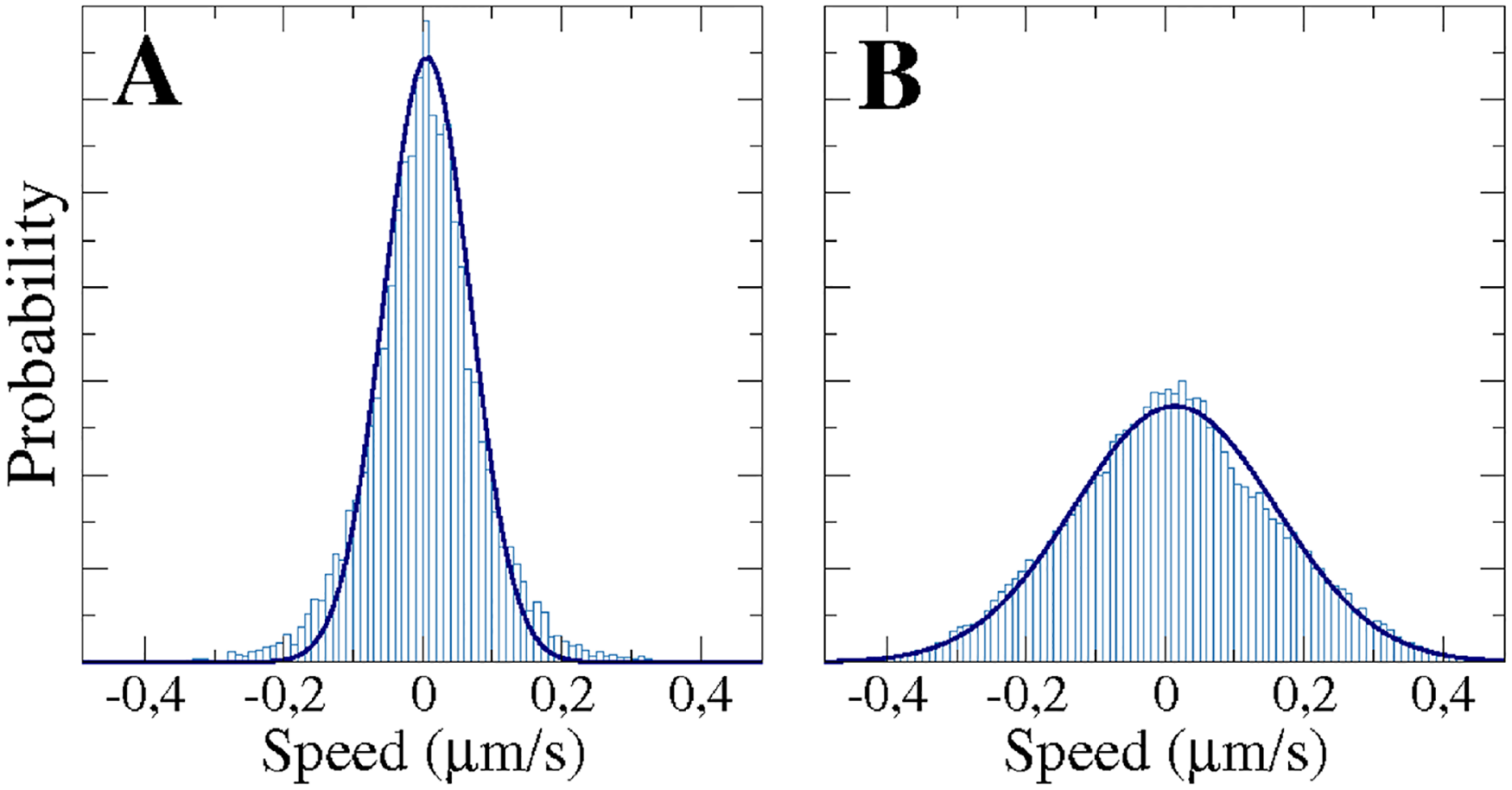
Histograms of the instantaneous velocity component *v*_*x*_ and the corresponding Gaussian fits (Solid lines) expected for Brownian motion. Results of large computer simulations corresponding to: *k*_*a*_ = 2 (10000 *s*) (A), and *k*_*a*_ = 5 (50000 *s*) (B).

In Fig 6, we show the distributions of the instantaneous velocity component *v*_*x*_ obtained from long-term computer simulations for two different values of the parameter *k*_*a*_. The distributions are centered at zero and are of non-Gaussian shape. A Gaussian best fit, displayed together with the distributions in Fig 6, systematically underestimates the probabilities of velocities around 0 and in the tails. Note that this effect is more pronounced for small values of *k*_*a*_ and is most likely an effect of the short sampling time scales [55]. The distributions of the perpendicular component *v*_*y*_ are identical and can be seen in S1 Fig.

Earlier statistical analyses of experimental trajectories have demonstrated that the distributions of the instantaneous velocity components of motile *Dictyostelium* cells are indeed non-Gaussian [16], which can be seen as a typical signature of non-equilibrium processes such as active motion [57] and, in particular, cell motility [58]. Note that Gaussian distributions are recovered for larger sampling time scales [55]. In the present experiments, we do not have a sufficient amount of cell trajectories to obtain meaningful velocity distributions, see the experimental distributions in S1 Fig. This is because we imaged at a higher magnification compared to previous studies, in order to capture the dynamics of the cell shape that we will analyze in the following.

### Variability in cell shape dynamics

#### Local membrane displacements

**Fig 7:**
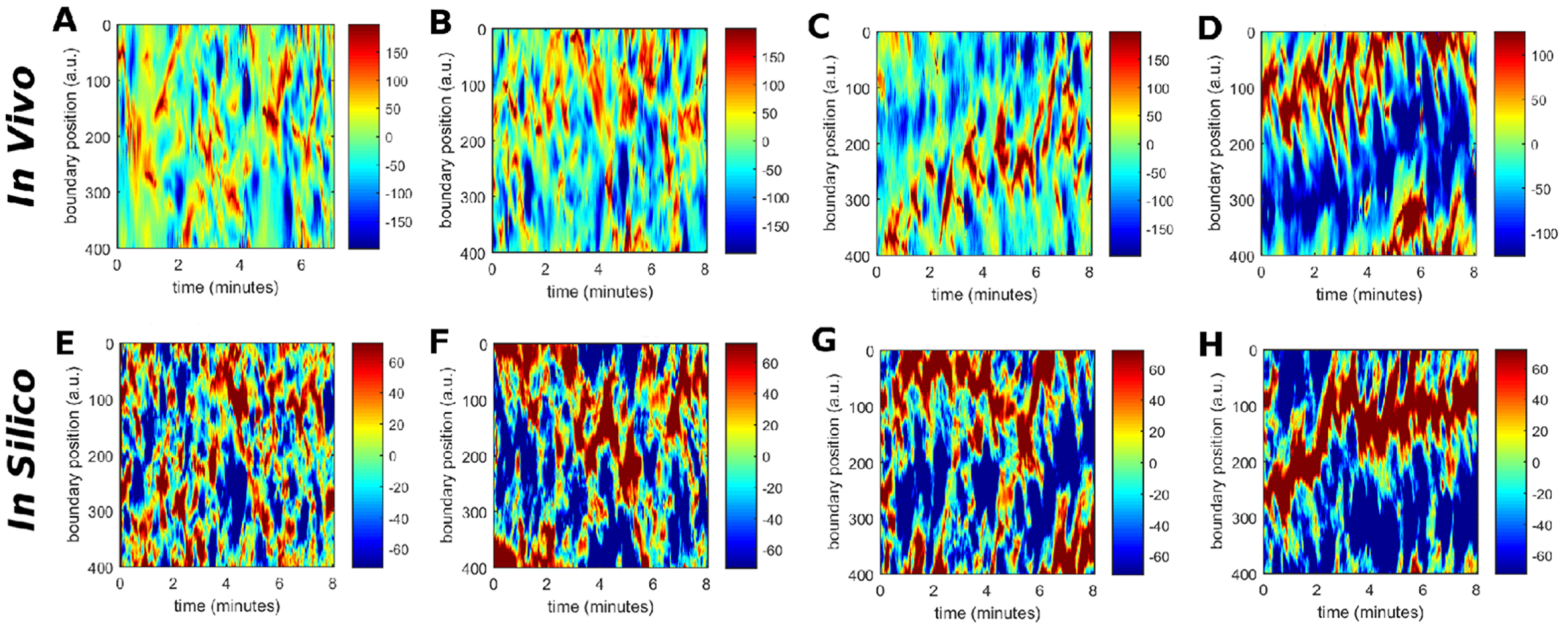
Comparison of the spatio-temporal evolution of the local displacement of the membrane with respect to its previous position *in vivo* (A-D) and *in silico* (E-H). The spatio-temporal evolutions *in vivo* correspond to cells with a speed of *v* = 0.09 *μm*/*s* (A), *v* = 0.12 *μm*/*s* (B), *v* = 0.15 *μm*/*s* (C), and *v* = 0.19 *μm*/*s* (D). The spatio-temporal evolutions *in silico* correspond to parameter values of *k*_*a*_ = 2 (E), *k*_*a*_ = 3 (F), *k*_*a*_ = 4 (G), and *k*_*a*_ = 5 (H).

The cellular membrane deforms during the process of cell migration, resulting in continuous changes of the cell shape. In both experiments and computer simulations, we determine the positions of the membrane elements every second and calculate their relative displacements with respect to the previous positions. Resulting experimental and computer-generated kymographs are shown in Fig 7(A)–7(D) and 7(E)–7(H), respectively. They show the local membrane displacement at 400 equidistant points along the cell perimeter over time.

For a slow moving non-polar cell shown in Fig 2(A), small membrane protrusions continuously appear and disappear at changing locations along the cell border. In this case, the cell does not move in a persistent fashion but rather hovers randomly around its initial location. This is reflected by small random displacements of the membrane over short times in all directions, see Fig 7(A). These experimental observations are qualitatively comparable with modeling results obtained for low values of the parameter *k*_*a*_ shown in Fig 7(E).

In contrast, panels (D) and (H) of Fig 7 correspond to a fast moving cell and a simulation with a higher value of *k*_*a*_ = 5, respectively. Here, the persistent direction of motion of the cell is reflected by displacements of the cell membrane that stably occur at the same position on the cell perimeter over more than 500 seconds. The displacements are positive in the front of the cell and negative at the back. Again, the results of both space-time diagrams, experimental and numerical, agree satisfactorily. In between these two limiting cases, the displacements and their persistence is systematically becoming more pronounced as the averaged velocity of the cell increases, i.e. for larger values of the parameter *k*_*a*_, resulting in more ballistic motion. The remaining panels in Fig 7 show these intermediate cases.

#### Cell elongation

**Fig 8:**
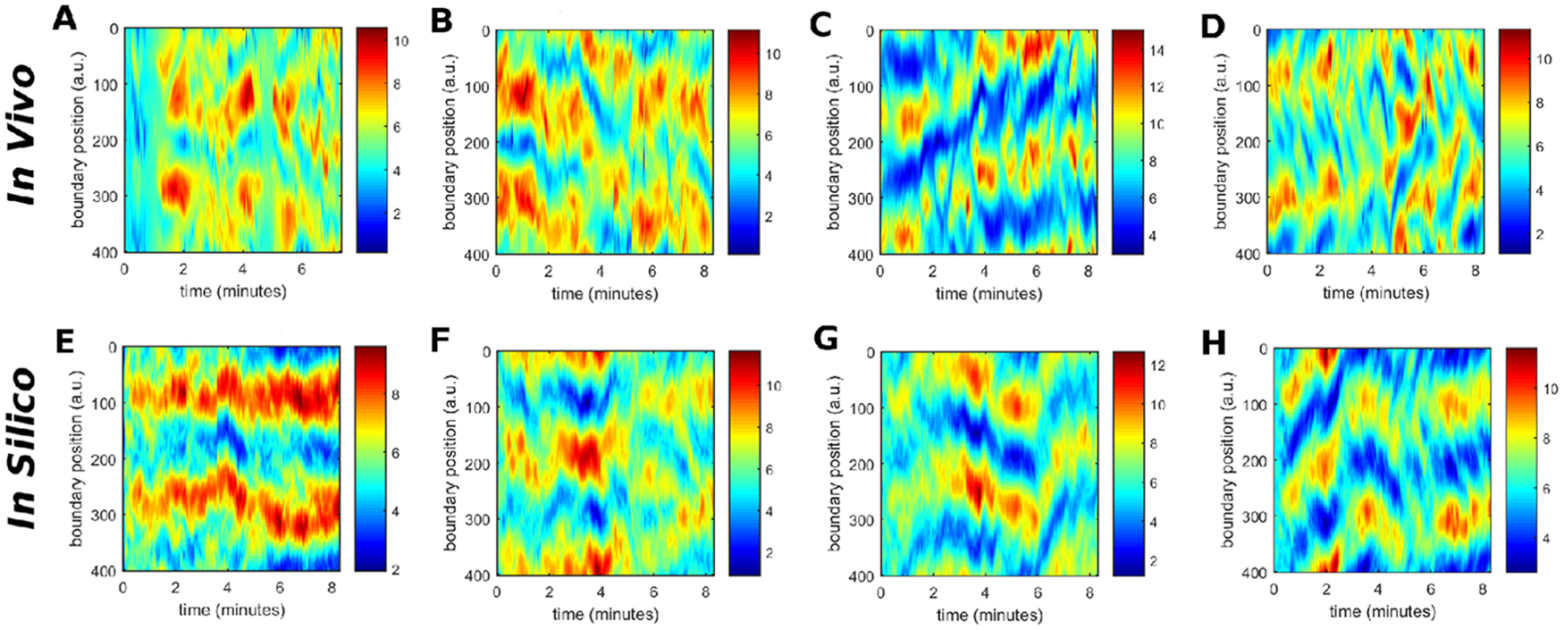
Comparison of the spatio-temporal evolution of the distance from the membrane to the centroid of the cell *in vivo* (A-D) and *in silico* (E-H). The spatio-temporal evolutions *in vivo* correspond to cells with a speed of *v* = 0.09 *μm*/*s* (A) *v* = 0.12 *μm*/*s* (B) *v* = 0.15 *μm*/*s* (C) and *v* = 0.19 *μm*/*s* (D). The spatio-temporal evolutions *in silico* correspond to parameter values of *k*_*a*_ = 2 (E), *k*_*a*_ = 3 (F), *k*_*a*_ = 4 (G), and *k*_*a*_ = 5 (H).

For the same set of cells, we have also quantified the elongation of the cell shape. Specifically, for each of 400 equidistant points along the cell membrane we determined the distance to the centroid of the cell. The temporal evolution of the distances between each point and the cell center is displayed as a kymograph in Fig 8. For a circular cell shape every point on the membrane would be at the same distance from the centroid and the corresponding space-time plot would be uniform. The color coding in Fig 8 reflects the deviations from the circular shape, with red (blue) regions being further away from (closer to) the center than the average. This shape parameter is related to the local displacements of the cell membrane, which was shown in Fig 7 above. However, while the elongation measure takes high values for the front and back parts of an elongated cell, the local displacement is positive at the front and negative at the back of the cell. This becomes particularly obvious when comparing panels (C,G) and (D,H) of Figs 8 and 7. In contrast, the distance to the centroid agrees closely with the profiles of the curvature, see S2 Fig, and can be seen as a local integration in space of the membrane curvature deformations.

#### Actin patterns

**Fig 9:**
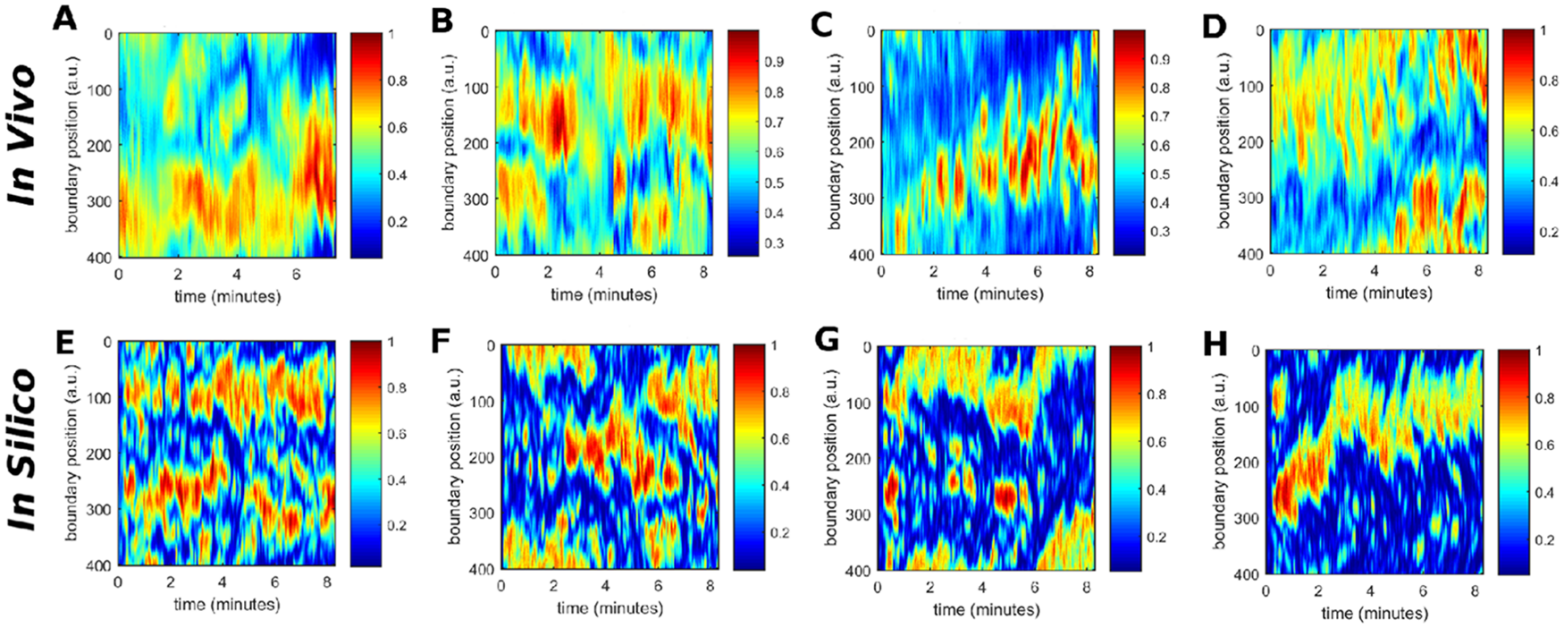
Comparison of the spatio-temporal evolution of the concentration of filamentous actin at the membrane *in vivo* (A-D) and the concentration of the biochemical component *c* at the membrane *in silico* (E-H). The spatio-temporal evolutions of the concentration of filamentous actin *in vivo* correspond to cells with a speed of *v* = 0.09 *μm*/*s* (A), *v* = 0.12 *μm*/*s* (B) *v* = 0.15 *μm*/*s* (C) and *v* = 0.19 *μm*/*s* (D). The spatio-temporal evolutions of the concentration of the biochemical component *c* *in silico* correspond to parameter values of *k*_*a*_ = 2 (E), *k*_*a*_ = 3 (F), *k*_*a*_ = 4 (G), and *k*_*a*_ = 5 (H).

The intracellular distribution of filamentous actin is shown in Fig 2 for two cells with different speeds, where the cell moving with higher velocity presents a more pronounced, persistent localization of filamentous actin at the front of the cell. In Fig 9(A)–9(D), we show kymographs of the concentration of filamentous actin along the cell membrane for four cells with different speeds. Two observations can be extracted from these figures. First, the persistent accumulation of actin at the front of fast, ballistically moving cells is confirmed and, second, regions of increased actin concentration co-localize with positive displacements of the cell membrane, compare Figs 9(A)–9(D) with 7(A)–7(D).

It is also remarkable that the actin patterns persist longer than the rapid pseudopods which appear and disappear on a faster time scale, compare Figs 9(A) and 7(A). This indicates that in the case of a fast moving cell, the accumulation of actin at the inner part of the membrane correlates with pseudopod formation and directed locomotion, whereas for slow moving cells, pseudopod formation is not confined to regions of increased actin accumulation and cell motion remains random.

The second part of Fig 9 displays the corresponding computer simulations, see panels (E-H). Here, the generic concentration *c* from Eq (6) of the model is shown, which exhibits more localized maxima in the concentration field compared to the experimentally observed actin patterns, which are smoother. The concentration *c* is not directly corresponding to the concentration of filamentous actin but can be regarded as an effective concentration that incorporates the dynamics of several signaling factors and cytoskeletal components that are involved in pseudopod formation. For example, it is known that increased concentrations of freshly polymerized actin co-localize with PIP_3_-rich membrane regions [51]. Therefore not more than a qualitative agreement of the generic concentration *c* with the experimentally observed actin patterns can be expected at this level of approximation.

## Discussion

In this article, we have introduced a mathematical model of amoeboid motility that was designed to capture the cell-to-cell variability in motile *Dictyostelium* cells by tuning the balance between polarity formation and intracellular noise. After introducing our model, we have presented a detailed comparison of experimental data from motile *Dictyostelium* cells and numerical simulations of the model. The cell trajectories, the evolution of the cell shape, and the actin-related intracellular concentration patterns generated by our model are in good agreement with the experimental observations. Even though the model produces a large variety of specific information, which can be compared with the experiments, it has a comparatively simple structure in comparison to other more complex biochemically inspired models that were previously presented. Specifically, our model combines a simplified biochemical dynamics in terms of a bistable reaction-diffusion equation, with a standard noise description based on the extensively studied Ornstein-Uhlenbeck process. In contrast to specific noise sources designed to produce the desired pseudopods and membrane deformations, we deliberately reverted to a very basic noise term and found this sufficient to produce realistic dynamics of intracellular patterns and cellular shape changes when combined with a dynamic phase field model of the cell shape. We will now compare our model with other models of *Dictyostelium* motility and discuss the differences in more detail.

The most basic models of *Dictyostelium* motility consider only the random motion of the center of mass of the cell and reduce the entire dynamics to stochastic ordinary differential equations for the motion of the cell center, see for example [16–18]. Such descriptions permit large realizations and the extraction of detailed statistical information about the random motion [59]. While a detailed stochastic analysis of our model trajectories is still missing, our first results on the non-Gaussian instantaneous velocity distributions show agreement with earlier analyses of experimental cell trajectories [16]. This is most likely an effect of our short sampling time scale. Using larger sampling intervals for the calculation of the velocities will result in an averaging effect and will eventually produce a Gaussian distribution [55].

On the other hand, many modeling efforts have focused on the intracellular dynamics of signaling patterns and cytoskeletal activity. For example, they consider the biochemical interactions at the cell membrane assuming a circular or spherical cell shape [30, 31, 60]. These models permit the study of the pattern formation mechanisms at the membrane of the cell with static borders. Waves, oscillations, and patches of the membrane concentrations are observed and the interactions with specific noise sources were studied [60]. Also the interior of the cell, together with the membrane, has been employed in the study of the intracellular pattern formation in circular cells [61] and in irregular cells generated with a static phase field [62].

An important improvement in the modeling of *Dictyostelium* motility was the coupling of the dynamics of membrane-bound intracellular concentrations patterns with cell shape deformations. Such coupling has been done with a membrane formed by finite elements coupled to biochemical reactions [63], and by the accommodation of the contour of the cell according to a one-dimensional dynamics of the biochemicals at the surface [9]. Also detailed combinations of biochemically inspired excitable and oscillatory signaling networks have been proposed to drive cell shape dynamics in models that also incorporate the viscoelasticity of the cell membrane [42, 43, 64].

The introduction of a dynamic phase field provides an elegant way to calculate also concentration patterns inside moving cell boundaries and not only restricted to the cell membrane. In recent years, this approach has become increasingly popular to model the motion of different cell types, such as fish keratocytes [38, 65], mammalian cells on micropatterns [66], or colliding cells in confined environments [67]. Also more detailed models of actin dynamics have been combined with the phase field approach. For example in [36], the local orientation of actin is taken into account and determines membrane deformations and movement of the cell. The phase field approach has also been applied to address specific aspects of amoeboid motility. In [32] a phase field was combined with a detailed model of phosphoinositide membrane signaling to analyze the impact of PIP_3_ waves on membrane deformation. In this model, a tailored noise source is introduced by assuming random events of PIP_3_ synthesis of a specific spatial extent. More recently, a complex description that combines actin and myosin dynamics with an activator representing the upstream signaling activity at the membrane was implemented together with a phase field to model *Dictyostelium* motility in open and confined environments [37]. This complex model comprises 28 parameters and was analyzed for a fixed setting that corresponds to a starvation-developed cell with a high velocity of *v* = 0.175 *μ*m/s. Also here, noise was introduced in a specific way, related to detailed modeling assumptions.

Unlike these earlier *Dictyostelium* models, we take a reductionist approach and propose a minimal model based on a bistable reaction-diffusion system that accounts for the intracellular biochemistry and a generic Ornstein-Uhlenbeck noise term. In this sense, our approach is more closely related to previous phase field models for keratocyte motility. However, unlike the keratocyte descriptions, our model captures the highly dynamic shape changes that are characteristic for pseudopod-driven amoeboid cell motion. In addition, our model has a unique parameter (*k*_*a*_) that encodes the cell-to-cell variability typically observed in experimental motility data from *Dictyostelium* experiments. By tuning this parameter, we consistently recover the variations observed in terms of velocity, polarity, and persistence of motion. We thus assume that *k*_*a*_ is constant but may take values that are different from cell to cell. The distribution of *k*_*a*_ values across a cell population is not prescribed by the model but can be inferred from our experimental data: as *k*_*a*_ is linearly related to the average cell speed (see Fig 5(C)), the distribution of *k*_*a*_ values is identical to the speed distribution shown in Fig 2(A), except for a constant factor. We may also interpret the parameter *k*_*a*_ as a measure for the degree of development of a cell and attribute variability in a population to an underlying heterogeneity in the developmental state of the cells. In this case, our model would encode the dependence of developmental progress on starvation time in a straightforward fashion, such that *k*_*a*_ = *k*_*ao*_ + Δ_*k*_ *t*, where Δ_*k*_ is different for each cell, explaining the behavioral variety in a population of *Dictyostelium* cells after four hours of starvation in our experiments. To account for cell-to-cell variability, we propose a random distribution of values of Δ_*k*_ that has to be chosen mimicking the experimental distribution of speeds shown in Fig 2(A). Note, however, that development does not necessarily progress in a linear fashion and that also de-differentiation effects may play a role, as they are known to have pronounced effects on the motility of *Dictyostelium* cells [68, 69]. Furthermore, our data indicates that *k*_*a*_ is not uniquely related to the developmental state but may also undergo more rapid changes, so that cells can switch between episodes of fast and slow movement, see S3 Fig.

Cell polarity in *Dictyostelium* is mediated by a number of typical cell front markers, such as activated Ras, PI3K and PIP3, that correlate with regions of increased actin polymerization and pseudopod formation. These signaling components localize at the cell front in response to a chemoattractant gradient. But they may also concentrate in self-organized dynamical patches at the cell membrane in uniform environments, see for example [70, 71]. Here, we proposed a model that combines a noisy bistable system to generate these dynamical signaling patches with a phase field description of a deformable cell boundary. A comparison of model simulations and live-cell recordings show that the model qualitatively reproduces the morphology of the cell shape and center-of-mass trajectories. Some aspects such as the speed correlation times even show quantitative agreement. The persistence time of the center of mass motion determined from our data in Fig 4D is smaller than previously reported values [54, 55]. This is most likely related to the different growth conditions. Potel and MacKay as well as Li et al. grew their cells on bacteria, whereas we used HL5 liquid growth medium (axenic growth), and it is well known, that the motility of *Dictyostelium* cells differs strongly between bacterially and axenically grown cells [68]. Indeed, Golé et al., who also used axenically grown cells, reported *τ*_*P*_ values closer to ours [56]. Our numbers are lower than the values reported by Golé et al., but they used the AX3-derived strain DH1, whereas we worked with AX2 cells, which may also affect the persistence times [54]. In summary our results thus demonstrate that essential features of amoeboid motion can be captured by a generic nonlinear mechanism for the formation of signaling patches in combination with a deformable domain. In particular, tuning the relative strength of patch formation versus noise reproduces the heterogeneity that is typically observed in a population of motile *Dictyostelium* cells.

Extensions of the model can be easily implemented to account for the chemotactic movement of *Dictyostelium* cells under external chemical gradients [72], or under confinement [73]. Furthermore, we may also envision modifications of the model that introduce more realistic biochemical reaction networks to recover the excitable dynamics observed in previous experiments [26] and explicitly include into our model the detailed dynamics of components that promote pseudopod formation, such as for example PIP_3_ and PI3K, as well as the antagonist concentrations PIP_2_ or PTEN as has been done in previous studies with more simplistic cell shapes [30–32, 60]. These extensions will be the focus of our future work.

## Supporting information

S1 Video *In vivo* motion of a cell with an average speed of *v* = 0.09 *μ*m/s. Red color is proportional to the concentration of actin. Some snapshots of the video are shown in Fig 2(A).
(AVI)

S2 Video *In vivo* motion of a cell with an average speed of *v* = 0.12 *μ*m/s. Red color is proportional to the concentration of actin.
(GIF)

S3 Video *In vivo* motion of a cell with an average speed of *v* = 0.15 *μ*m/s. Red color is proportional to the concentration of actin.
(GIF)

S4 Video *In vivo* motion of a cell with an average speed of *v* = 0.19 *μ*m/s. Red color is proportional to the concentration of actin. Some snapshots of the video are shown in Fig 2(C).
(AVI)

S5 Video *In silico* motion of a cell with an average speed of *v* = 0.09 *μ*m/s. Red color is proportional to the concentration c resulting from Eq (6). Some snapshots of the video are shown in Fig 2(B).
(GIF)

S6 Video *In silico* motion of a cell with an average speed of *v* = 0.12 *μ*m/s. Red color is proportional to the concentration c resulting from Eq (6).
(GIF)

S7 Video *In silico* motion of a cell with an average speed of *v* = 0.15 *μ*m/s. Red color is proportional to the concentration c resulting from Eq (6).
(GIF)

S8 Video *In silico* motion of a cell with an average speed of *v* = 0.19 *μ*m/s. Red color is proportional to the concentration c resulting from Eq (6). Some snapshots of the video are shown in Fig 2(D).
(GIF)

S1 Fig Histograms of the velocity distribution for both component of the velocity *v*_*x*_ (blue) and *v*_*y*_ (red) and the corresponding Gaussian fits (Solid lines) expected for brownian motion. Results for large (10000 *s*) computer simulations corresponding to: *k*_*a*_ = 2 (A), *k*_*a*_ = 3 (B), *k*_*a*_ = 4 (C) for 10000 s, and *k*_*a*_ = 5 (D) for 50000 s; and for regular (500 *s*) experiments, corresponding to cells with average velocity: 0.09 *μm*/*s* (E), 0.12 *μm*/*s* (F), 0.15 *μm*/*s* (G), and 0.19 *μm*/*s* (H).
(EPS)

S2 Fig Comparison of the spatio-temporal evolution of the local curvature of the membrane *in vivo* (A-D) and *in silico* (E-H). The spatio-temporal evolutions *in vivo* correspond to: *v* = 0.09 *μ*m/s (A), *v* = 0.12 *μ*m/s (B), *v* = 0.15 *μ*m/s (C), and *v* = 0.19 *μ*m/s (D). The spatio-temporal evolutions *in silico* correspond to: *k*_*a*_ = 2 (E), *k*_*a*_ = 3 (F), *k*_*a*_ = 4 (G), and *k*_*a*_ = 5 (H).
(EPS)

S3 Fig Variability in the motion pattern of a single cell. Example of a cell that switches from a slow moving state with only little net displacement to a state of rapid persistent motion.
(EPS)
