# Supplementary figures and images for: Modeling random crawling, membrane deformation and intracellular polarity of motile amoeboid cells
Source: PLoS One. 2018 Aug 23;13(8):e0201977. doi: 10.1371/journal.pone.0201977 (PMC6107139; doi:10.1371/journal.pone.0201977)

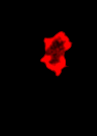

Supplement: S2 Video — Red color is proportional to the concentration of actin. (GIF) [file pone.0201977.s002.gif]

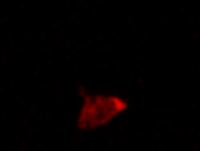

Supplement: S3 Video — Red color is proportional to the concentration of actin. (GIF) [file pone.0201977.s003.gif]

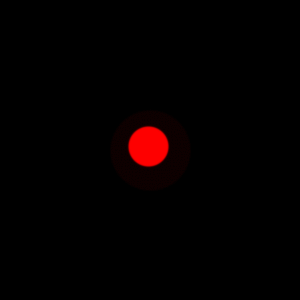

Supplement: S5 Video — Red color is proportional to the concentration c resulting from Eq (6). Some snapshots of the video are shown in Fig 2(B). (GIF) [file pone.0201977.s005.gif]

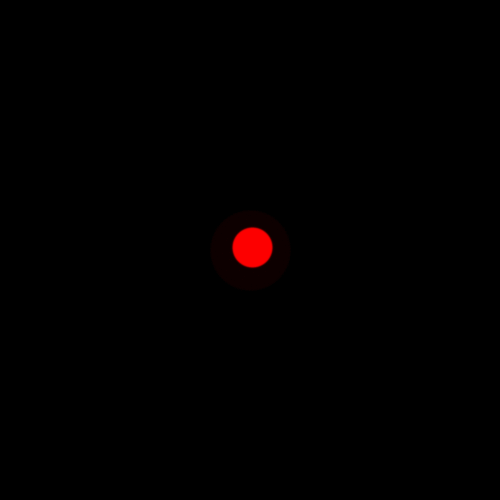

Supplement: S6 Video — Red color is proportional to the concentration c resulting from Eq (6). (GIF) [file pone.0201977.s006.gif]

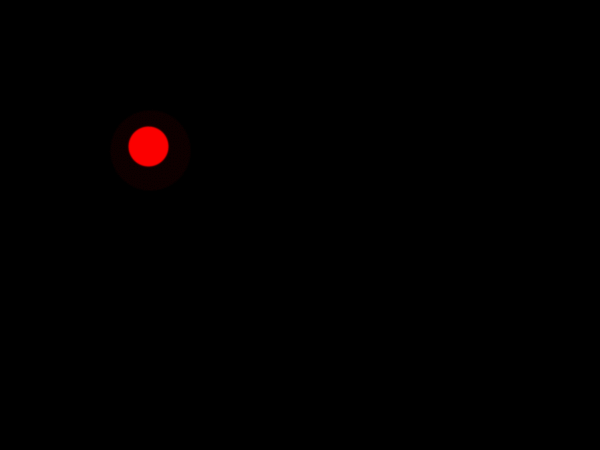

Supplement: S7 Video — Red color is proportional to the concentration c resulting from Eq (6). (GIF) [file pone.0201977.s007.gif]

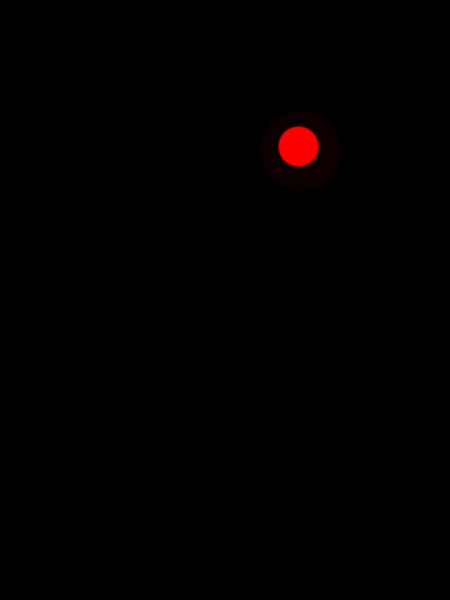

Supplement: S8 Video — Red color is proportional to the concentration c resulting from Eq (6). Some snapshots of the video are shown in Fig 2(D). (GIF) [file pone.0201977.s008.gif]
